# The Antiseptic Value of Chloretone

**Published:** 1905-11-15

**Authors:** T. A. Gormly

**Affiliations:** Mount Vernon, Iowa


### THE ANTISEPTIC VALUE OF CHLORETONE.

BY T. A. GORMLY, D.D.S., MOUNT VERNON, IOWA.

Four or five years' use of chloretone has demonstrated the practical utility of an alcoholic solution for topical application previous to the use of the hypodermatic needle in the gums. A ten or twenty percent solution in seventy-five percent alcohol may be used, and unlike cocain causes no toxic effect. An application for this purpose should have the following qualities: 1st, detergent; 2d, antiseptic; 3rd, anesthetic. The alcohol possesses the first requirement, cutting the mucous and leaving the membrane absolutely clean. The tissue is then readily acted upon by the antiseptic agents, alcohol and chloretone, and sterilization of the field of operation results. Finally the anesthetic action of the chloretone is had on the tissues and the hypodermatic needle can be used with a minimum of pain and no possibility of septic infection.

